# Miniaturized Integrated Platform for Electrical and Optical Monitoring of Cell Cultures

**DOI:** 10.3390/s120811372

**Published:** 2012-08-21

**Authors:** Carmen Moldovan, Rodica Iosub, Cecilia Codreanu, Bogdan Firtat, Daniel Necula, Costin Brasoveanu, Ion Stan

**Affiliations:** 1 National Institute for Research and Development in Microtechnologies, 126 Erou Iancu Nicolae, Bucharest 077190, Romania; E-Mails: rodica.iosub@imt.ro (R.I.); cecilia.codreanu@imt.ro (C.C.); bogdan.firtat@imt.ro (B.F.); daniel.necula@imt.ro (D.N.); costin.brasoveanu@imt.ro (C.B.); 2 Romelgen SRL, Ion Berindei 11, Sector 2, Bucharest 077190, Romania; E-Mail: stan.ion2007@gmail.com

**Keywords:** integrated microsensors and microelectrodes, optical and electrical testing, miniaturized platform, cell culture monitoring

## Abstract

The following paper describes the design and functions of a miniaturized integrated platform for optical and electrical monitoring of cell cultures and the necessary steps in the fabrication and testing of a silicon microchip Micro ElectroMechanical Systems (MEMS)-based technology for cell data recording, monitoring and stimulation. The silicon microchip consists of a MEMS machined device containing a shank of 240 μm width, 3 mm long and 50 μm thick and an enlarged area of 5 mm × 5 mm hosting the pads for electrical connections. Ten platinum electrodes and five sensors are placed on the shank and are connected with the external electronics through the pads. The sensors aim to monitor the pH, the temperature and the impedance of the cell culture. The electrodes are bidirectional and can be used both for electrical potential recording and stimulation of cells. The fabrication steps are presented, along with the electrical and optical characterization of the system. The target of the research is to develop a new and reconfigurable platform according to the particular applications needs, as a tool for the biologist, chemists and medical doctors working is the field of cell culture monitoring in terms of growth, maintenance conditions, reaction to electrical or chemical stimulation (drugs, toxicants, *etc.*). HaCaT (Immortalised Human Keratinocyte) cell culture has been used for demonstration purposes in order to provide information on the platform electrical and optical functions.

## 1. Introduction

For centuries doctors and biologists have dedicated significant efforts to investigate diseases, to analyse the effects of drugs and to identify the best solutions for improving the human health condition. If for many years the testing needed for validation of drugs, to study their toxic effects, and for monitoring the evolution of several diseases (e.g., cancer) has been extensively done on animals, in the last two decades the replacement of animal studies by studies on cell cultures has been recommended more and more. The European laws and regulations regarding experimentation on animals are being taken into consideration. Directive 86/609/EEC stipulates “the approximation of laws, regulations and administrative provisions of the Member States regarding the protection of animals used for experimental and other scientific purposes” while the Directive 2000/0077 (COD) on the laws of the Member States relating to cosmetic products, has among its objectives: “to prohibit the testing of cosmetic products on animals” and “to prohibit the testing of cosmetic ingredients on animals and the marketing of cosmetics tested on animals or containing ingredients tested on animals as soon as alternative testing methods have been validated by the Commission”.

Cell culture technology has thus seen a huge expansion and the tools for using it are more and more accessible to biological laboratories. There are strong recommendations, at least at the European level, to use cell culture as a tool for investigation and simulation of organs, and beings' living conditions when exposed to external stimulation like chemicals (drugs, toxicants), electricity (potential, current), radiation (electromagnetic field, nuclear, X-ray, ultraviolet radiation) and others.

Bionanotechnology has become the modern tool for integrating biomaterials research with nanomaterials features providing new research and developments in biomedical field. New tools and apparatus are needed for empowering biomedical research by offering increased functionality, high sensitivity, easy handling, user friendly interfaces and cost reduction.

The focus of our paper is the development of a miniaturized platform providing cell culture monitoring, recording and stimulation by using integrated microsensors and microelectrodes fully connected to the computer for measurements and data acquisition. An optical module based on a video camera and its dedicated software, connected to the computer, allows the user to take pictures and store them on the computer. The clarity and magnification of this video camera can be appreciated as modest but the pictures are comparable with the optical pictures that can be found in literature and the method has the great advantage of easy and safe manipulation (the cells need not be taken out from the incubator and placed under a microscope, as usually happens in biomedical laboratories).

In 2009 Ming Ni *et al.* [1] presented a review on MEMS platforms for cell culture that highlighted the importance of MEMS technologies for cell culture research. The technologies and devices discussed therein were mostly related to microfluidics for biomedical applications. Several sensors and electrodes were also reviewed and the advantages of MEMS platforms in terms of “cost-effectiveness, controllability, low volume, high resolution, and sensitivity” were presented.

Chang *et al.* developed a MEMS-based dynamic cell-to-cell culture platforms using electrochemical surface modifications [2] employing ITO miniaturised electrodes on Pyrex substrates. The platform has been used to demonstrate dynamic cell-to-cell experiments of NIH 3T3 fibroblasts and Madin Darby canine kidney cells. The authors considered such a platform to be a basic “versatile tool to characterize transient cell-to-cell interactions”, extending the goal of their platform and aiming to provide a more general tool.

The task of providing tools for cell culture recording and manipulation has also entered the industrial application area. The US patent “Robotized platform for cell culture in miniature reactor” [3] deals with the approach of having a robotic arm with sensors for measuring the optical parameters of cell cultures placed in several wells.

There are numerous scientific and innovative approaches arising from the need to have automatic easy to use platforms, and involving automation for cell culture monitoring, handling, recording or stimulating. That is the context of our research and the specific functionality of the platform we have developed arises from addressing the needs of biomedical research laboratories.

Numerous electrical devices have been developed over the years for interacting with living bodies or cells, *in vivo* and *in vitro*. By using MEMS technology a lot of advantages in terms of miniaturization, high reproducibility, high sensitivity, biocompatible materials, low cost and others need to be taken into consideration. MEMS technology can provide a large variety of microsensors, able to work with very small quantities of liquid, can provide microfluidic chips and also full automation, data acquisition and signal processing. The silicon microchip we developed in 2002 [4] integrated eight electrodes for recording and stimulation of neuronal cells. At that moment it fulfilled the needs of neurophysiologists for investigating the nervous system using state of the art microfabrication technology, allowing fabrication of multiple microelectrode arrays for simultaneously recording and stimulating a large number of neurons in the brain [5]. The applications of these electrodes require implantable devices able to interface technical aids with peripheral nerves, and the brain [6]. They have been used to perform fundamental neuroscientific studies and some of them have been transferred into clinical practice in diagnosis, therapy and rehabilitation, respectively. A bidirectional information transfer is necessary, either to record bioelectrical signals of a nerve or muscle or to perform nerve and muscle excitation by electrical stimulation. The objectives of neural implants as presented by [7] are either the detection of functions in diagnosis and offers adequate tools, methods, and materials but so far, no MEMS-based active medical device has been transferred into clinical practice.

Using the MEMS technology and our previous experience in microelectrodes for neuronal cell monitoring we are now going to expand the silicon microchip functions, making it able to monitor pH, temperature, impedance and potentials for many other applications. In the present paper we will call this silicon microchip a “microprobe”. The monitoring of cell culture in different media, growth control, lifetime, and cell behaviour when exposed to toxicants using electrodes [8,9] and miniaturized sensors is a new research direction approached equally by engineers and biologists. The common work of engineers, biologist and medical doctors show an increased need for miniturized, easy to manipulate, computer controlled devices and platforms integrating more and more functions in order to facilitate laboratory research and the applications development.

**Figure 1.**
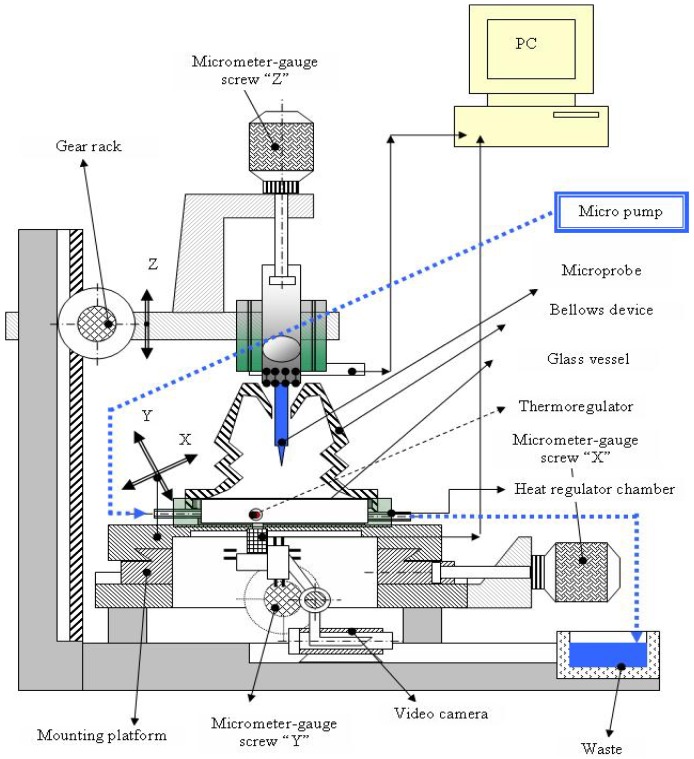
Scheme of the integrated platform.

The need of an integrated platform with electrical and optical functions, fully controlled by the computer, allowing 24/7 monitoring for medical research purposes, toxicology tests, pharmaceutical and cosmetics industry tests, is increasingly high, and this was the reason behind the present research work. The integration of electrical and optical devices on the platform is realized according to the following scheme (Figure 1) [10].

The platform contains: (a) one micromachined microprobe hosting on the shank two impedimetric sensors, 10 platinum electrodes, two pH electrodes and one temperature sensor and allowing wire bonding between the pads and the PCB dedicated to *electrical* measurements; (b) a video camera connected to the computer allowing on-line picture acquisition for *optical monitoring*; (c) a miniaturized incubator for growing cells; (d) a microfluidic system directly connected to the incubator allowing insertion of media and samples onto the cell culture; (e) packaging and connections between sensors and camera and the computer; (f) software for on-line data acquisition. The microprobe providing all electrical measurements can be used *in vitro* or *in vivo*. Initially developed for neuronal cell investigation, the microprobe has been used for both recording and stimulation. Later on, the microprobe has been used with other types of cell culture and became a “general tool” providing electrical measurements. The integration of the microprobe into one platform has the goal of offering a complete system for cell culture monitoring with applications in medical research, pharmaceutical industry, toxicity monitoring, *etc.* The platform compounds can be changed, modified or replaced. We can thus speak of a platform reconfigurable according to the application needs. The silicon microchip can be modified for integration of more sensors, the optical module can be modified by adding a fluorescence detector and so on.

## 2. Experimental

The platform functions and the experiments performed by using it will be presented in this section.

### 2.1. Electrical Function. Design and Fabrication Technology

**Figure 2.**
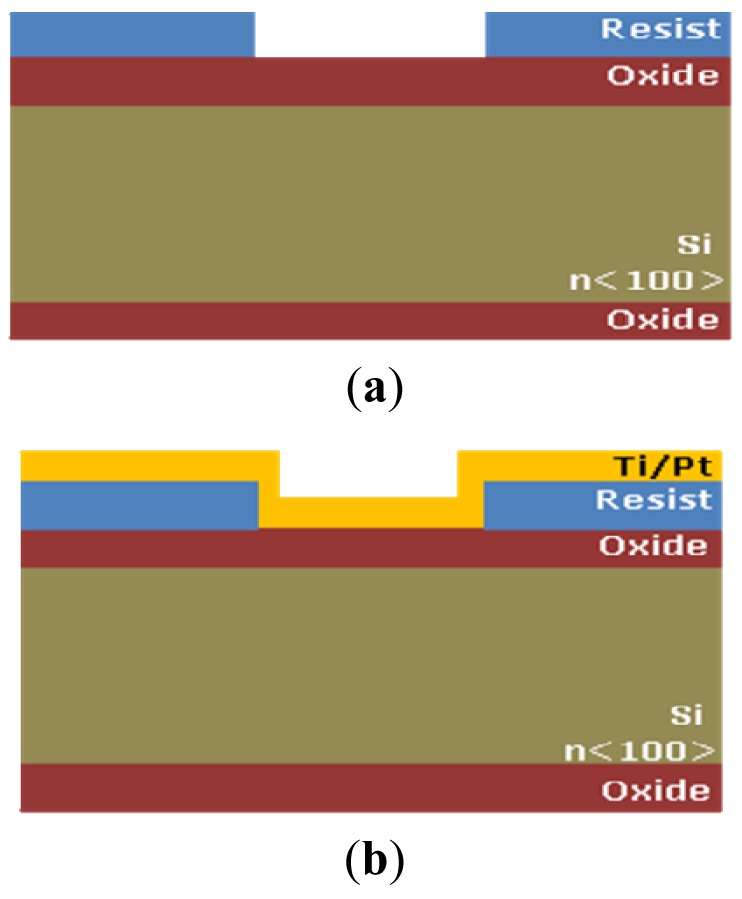

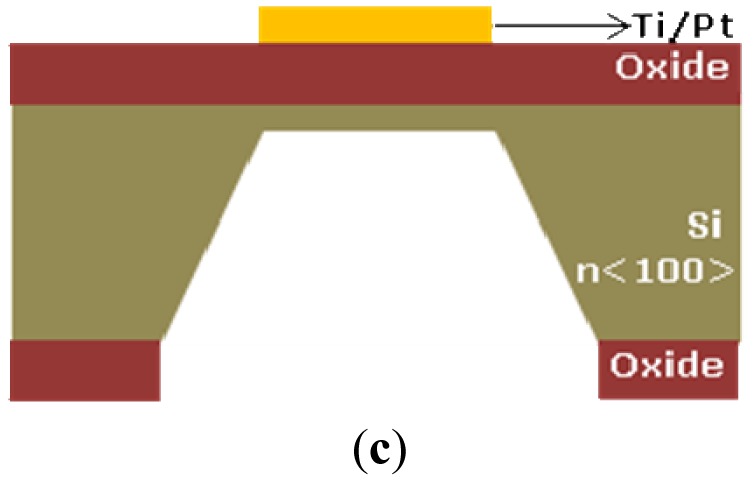
Silicon microprobe fabrication steps, cross-section.

The electrical function of the platform is performed by the microprobe described before. All sensors are integrated on the microprobe shank and are realized by unitary process flow microtechnology. The microprobe fabrication steps are presented below and are represented in Figure 2:

– Silicon wafers, n<100>, ρ = 3 Ωcm
– Silicon oxidation, x_ox_ = 1,6 μm [Figure 2(a)]
– Resist Spin coating t_resist_ = 4 μm
– Photoresist patterning (UV, E = 100 mJ/cm^2^, t_dev_ = 30 s), Mask 1 [Figure 2(a)]
– Ti/Pt 15 nm/200 nm deposition [Figure 2(b)]
– Photoresist removal [Figure 2(c)]
– Si_3_N_4_ deposition and patterning for opening contact windows between cells and electrodes and the opening the interchip for allowing the silicon anisotropic etching from the front size. Mask 2 [Figure 2(c)]
– Backside oxide patterning and anisotropic etching for microprobe release and thinning [11,12], t_etching_ = 400 min, T = 80 °C in TMAH, Mask 3 [Figure 2(c)]

**Figure 3.**
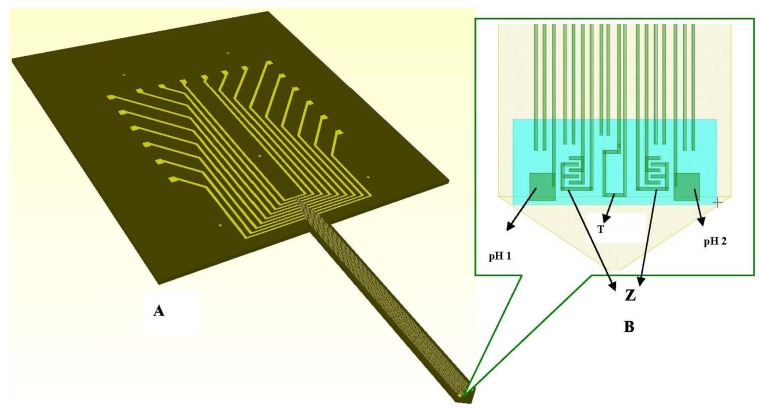
Silicon microprobe design (**A**) and details of the shank (**B**) resize picture.

Considering the technological steps needed to achieve the sensors integration we designed the “electrical microdevice” called microprobe using the COVENTORWARE software package for pattern generation. The scheme of the microprobe and the layout are presented in Figure 3.

The layout of the microprobe is transferred on chromium masks, three masks being needed for full fabrication of the microprobe. The masks are used to transfer the geometries into the resist on silicon wafer allowing selective etching of layers deposited on the silicon wafer and of silicon itself. The long and thin shank is designed to be inserted into small liquid cavities, provoking minimum damage to the monitored cells and leaving a larger space in the incubator for observation of cells and obtaining clear pictures via the video camera.

**Figure 4.**
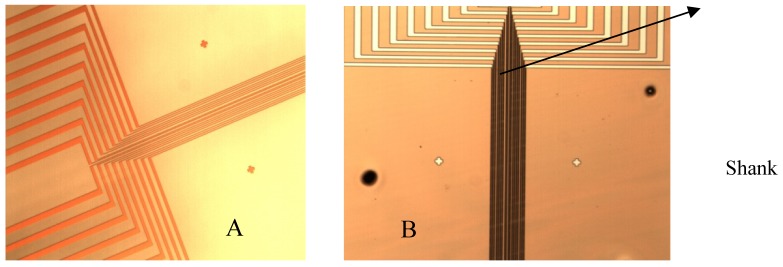
Fabricated microprobe details.

The ten electrodes of 4 μm width and 3 mm length each, one temperature sensor, two pH working electrodes and two interdigitated based (IDT) impedimetric sensors are patterned on the shank and connected to pads (100 μm × 100 μm each) spread on an enlarged area for wires bonding and manipulation (Figure 4(A)). Details of the electrodes on the shank and enlarged area are presented in Figure 4(A,B).

The platinium electrodes have been manufactured in two versions: (1) Pt electrodes on SiO_2_ substrate; and (2) by nanostructuring of SiO_2_ substrate and patterning Pt electrodes on top for increasing the electrode area; a femtosecond laser have been used for this purpose and the laser nanostructuring process has been performed by the National Institute of Laser, Plasma and Radiation in Romania. The electrodes are bidirectional.

They can be used for recording, but also for stimulation. The difference comes from the electronics. The recording part means recording electrode potentials and in our case storing the data on the computer while the electrical stimulation part means sending small electrical currents into the cell culture and in our case monitoring the electrical and optical effects. The electronic module and the electrode connections allow both functions: recording and stimulation.

### 2.2. The Temperature Sensor

The temperature of the cell culture depends on the type of cell line under study. Usually the recommended temperature is 36–37 °C but different temperatures can be required [13,14] depending on the type of cell culture. The incubator is normally set-up for the required temperature. Our mini-incubator can work between 27 and 40 °C. A platinum probe is inserted in the mini-incubator vessel and is connected to the thermostat. However, for precise temperature measurement, an integrated temperature sensor is placed on the silicon microprobe shank and is measuring the temperature inside the cell culture, being in direct contact with the cells. The temperature sensor is a miniaturized platinum resistor having the ability to measure the temperature and to control it by a computer controlled electronic feedback and it is designed to have a sensitivity of S = 0.4 Ω/°C and a precision of ±0.2 °C. The temperature sensor has been calibrated around 37 °C, which is the working temperature of the system cell monitoring. The temperature sensor is 6 mm long and 4 μm width. The sensor is a miniaturized platinum resistor having the ability to measure the temperature and to control it by a computer controlled electronic feedback. If the temperature is too low, the command from the computer is a current injection increasing the platinum resistor temperature and increasing the fluid surrounding temperature.

The experiments for temperature sensor validation have been performed into the mini-incubator, which has the capability to set-up the needed temperature and to measure it by using a separate Platinum probe integrated into it. The microprobe shank from Figure 3 has been inserted into the aqueous solution inside the mini-incubator vessel.

### 2.3. pH Sensor

pH measurement in cell culture is an important indicator of the cell growth phase [14,15]. pH variations during the cells' growth, maturity and death phases depends on the particular type of cell culture, media, air composition and temperature conditions [15,16]. The developed pH sensor works properly over a large pH domain (pH 4.0 ÷ pH 7.5), covering very well the needs of cell culture monitoring [17].

**Figure 5.**
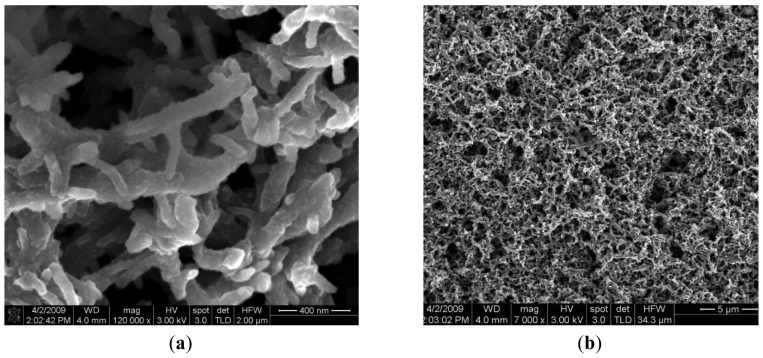
SEM pictures of the polyaniline conductive layer deposited on Ti/Pt electrode. (**a**) the nanofibers aspect of the polyaniline; (**b**) large area and porous aspect of 25 microns thick polyaniline.

The reference electrode (Ag/AgCl, KCl 3 M) is a silver wire inserted into a small PDMS cavity filled with AgCl/KCl 3M. The sensor measurement is a voltage measurement at zero current. The instrument has an input resistance higher than 1 GΩ. The voltage is measured between two electrodes: the active electrode and the reference electrode. The measurement cell is calibrated individually based on the pair of values: voltage and pH. Two pH working electrodes are placed on the microprobe shank (Figure 3(B)). The pH working electrodes are integrated and planar. The pH working electrode of the pH sensor is a solid state sensor based on a conductive polymer, deposited on a platinum patterned microelectrode, developed on a silicon silicon wafer with thermal SiO_2_ on top, followed by a lift–off process for patterning the Ti/Pt metallic layer of the electrodes. The Ti/Pt electrode is covered by Si_3_N_4_ which is patterned by optical lithography followed by RIE etching of Si_3_N_4_ leaving openings where the polyaniline polymer is needed to be deposited [Figure 2(c)]. The next step is the electrochemical deposition of polyaniline conductive emeraldine base form on top of Ti/Pt. The working electrode is formed by Ti/Pt with conductive polyaniline on it. The electrochemically deposited polyaniline has an intrinsic nanofibre structure of 100 nm diameter, as we can see in Figure 5(a) [12].

Polyaniline is a material sensitive to hydrogen ions and additionally a polyaniline coated platinum surface has a much larger area than simple uncoated platinum, which enhances the exchange current density. The advantage of obtaining a larger electrode area exposed to the solution to be measured in the conditions of a limited space on the microprobe shank are obvious. Figure 5(b) shows the fibrous and porous aspects of polyaniline deposited on Ti/Pt.

**Scheme 1.**
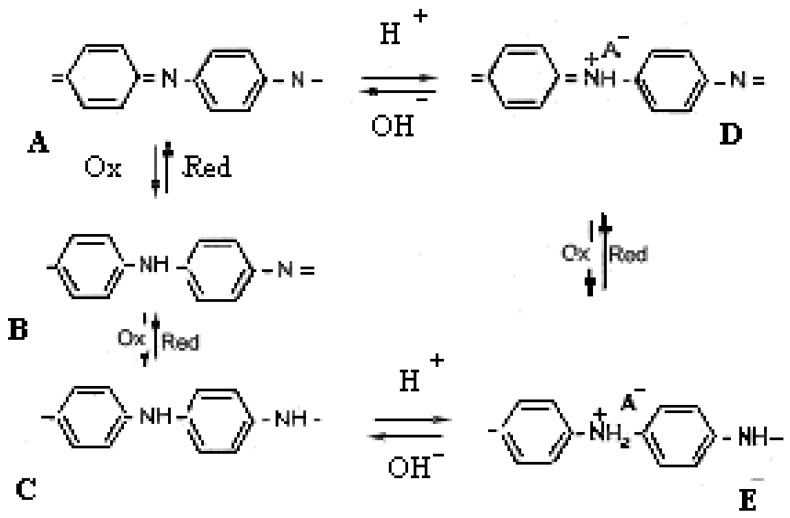
The redox states of polyaniline.

The structure of the undoped polyaniline may vary between two limit structures [11]: the completely oxidized state, called pernigraniline, violet and insulating (A) and the completely reduced state, called leucoemeraldine, yellow and insulating (C). The redox states of polyaniline are shown in Scheme 1. The emeraldine salt form (Scheme 1, E) is the conductive polyaniline form and it is a protonated state. The emeraldine salt is highly conductive, green color and can be directly obtained by oxidative polymerization of aniline in a HCl environment. If stronger oxidation agents are used the E state is turned into protonated pernigraniline (salt), which is supposed to be conductive too. Continuing the basic treatment (NH_4_OH) leads to pernigraniline base (A), so the reversible interconversion of structures A, B, C, D, E requires an acid/base couple or an oxidant/reducer couple or both. The oxido/reduction reaction may be performed electrochemically.

The deposition of conductive polyaniline using Voltalab 10 is done by the following steps: (a) aniline solution preparation; and (b) emeraldine salt deposition on the cathode, described below:

- The used electrolyte solution contains the following components: aniline, C_6_H_5_NH_2_ 1 M (99%, Mw = 93.13 g/mol; ρ = 1.0217 g/mol) and perchloric acid, HClO_4_ 1 M (60%, Mw = 100.46 g/mol; ρ = 1.67 g/mol), which is the dopant. The monomer/dopant (aniline/perchloric acid) ratio was 1:1 and 1M aniline perchlorate solution will be obtained with pH∼1, with a small excess of dopant, perchloric acid ∼2% to avoid precipitating the aniline. Anodic oxidative polymerization of aniline was performed at ambient temperature and the electrolyte also had the role of aniline dopant.
- The emeraldine salt on the substrate of the gold electrode was obtained with three cycles of deposition by the Potential Cyclic Voltammetry method with the following program: scanning speed 100 mV/s.; potential for polymerization of aniline = 1,000 mV/s, and the time of the scanning for one cycle = 62 seconds.

### 2.4. Sensor Connection

**Figure 6.**
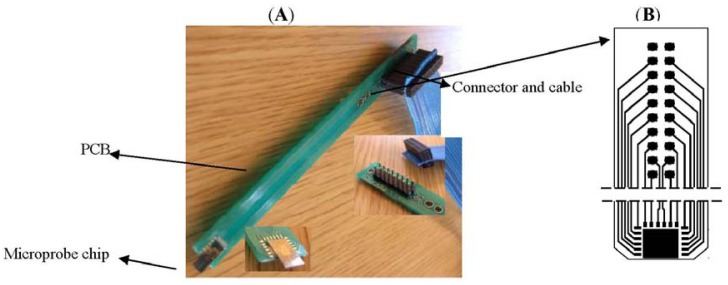
Assembled microprobe on a PCB (**A**) and PCB layout (**B**) center figure.

The electrodes and sensors for cells monitoring placed on top of the microprobe shank are designed to be inserted into the media (*in vitro* or *in-vivo*) [18]. The silicon microprobe is assembled on a printed board as shown in Figure 6, designed to accommodate the microprobe chip assembly and connection of sensors to electronic modules and PC and allowing the easy manipulation with minimum cells damage. The microprobe uses the wire bonding technique for connecting the electrodes to the outside world (electronics). For easy interfacing of the PCB board with the command electronics and measurement instruments standard microconnectors and ribbon cables have been used (Figure 6). In addition to robustness and versatility, this solution ensures the accuracy and precision of measurements by minimization of the leakage and noise components induced by connections.

### 2.5. Mini-Incubator

The mini-incubator contains a glass vessel, a polymeric bellows device and a thermostat. The glass vessel has been designed and manufactured with several special features: two openings for micro-fluidic inlet and outlet, one opening for gas insertion, and one opening for external CO_2_ sensor insertion. Inside the vessel, a 500 μm width cavity is provided for insertion of a platinum wire acting as temperature sensor and connected to the mini-incubator thermostat. This sensor controls and regulates the temperature inside the mini-incubator for keeping the cells at 37 ± 0.2 °C. The air inside the mini-incubator has 5% CO_2_. The vessel has 4 cm diameter resulting in a surface of 12.56 cm^2^ and a volume of ∼6 cm^3^.

The following components of the platform will be submitted to the sterilization process: the mini-incubator vessel, the mini-incubator bellows device (cap) and the microprobe. The sterilization process took place under a laminar flow hood. The sterilisation steps proposed for the mini-incubator hosting the cell culture and for the microprobe which will be inserted into the cell culture, monitoring the electrical parameters are:

- Placing the compounds to be sterilised into an ultrasonic tank for 5 minutes, filled with a solution with good detergent properties, like quaternary ammonium compounds; The procedure is repeated for another 5 minutes;
- Cleaning in deionized water inside the ultrasonic tank, twice for 5 minutes;
- Cleaning in H_2_SO_4_ − H_2_O_2_ = 7:3, 5 minutes, at room temperature, twice, The sterilization process uses an aqueous solution of hydrogen peroxide (H_2_O_2_) with a concentration of 30 to 35% to achieve the germ-killing effect in combination with sulfuric acid 98%;
- Cleaning in deionized water 5 minutes, twice;
- Drying two hours in a thermostatic box at 120 °C under nitrogen flow;
- Cool at room temperature and put the media dedicated to cell culture growing in the incubator vessel, under UV lamp;
- At that moment the mini-incubator is prepared for cell culture. The cell culture prepared in advance according to its specific protocol is injected into the vessel using a pipette. The bellows device is fixed afterwards on the mini-incubator vessel and the microprobe is inserted inside the incubator, penetrating the cell culture by 3 mm.

Covering the vessels with the bellows device, inserting the microprobe and closing all with a special button, makes the mini-incubator ready to use. The platform is set-up by now for cell culture investigation. HaCaT cells have been utilized due to their high capacity to differentiate and proliferate *in vitro* [19] and have been grown in 1.6 mM calcium.

## 3. Results and Discussion

### 3.1. Integrated Platform

The miniaturized integrated platform developed for electrical and optical monitoring of cell culture has the following important characteristics:

- Development of a miniaturized incubator, sterilized and precisely controlling the temperature inside (around 37 °C).
- Controlling CO_2_ inside the mini-incubator through a sensor external to the microprobe.
- Microfluidic system for cell culture feeding and circulation of fresh media.
- Electrical and optical monitoring by: (a) interfacing the microprobe containing all integrated sensors with the computer; (b) *in-situ* temperature sensor interfaced with the computer for additional control of temperature inside the incubator; (c) optical data acquisition by interfacing a video camera with the computer; (d) implementation of a LabVIEW-based Graphical Interface for all measurements and data acquisition.

**Figure 7.**
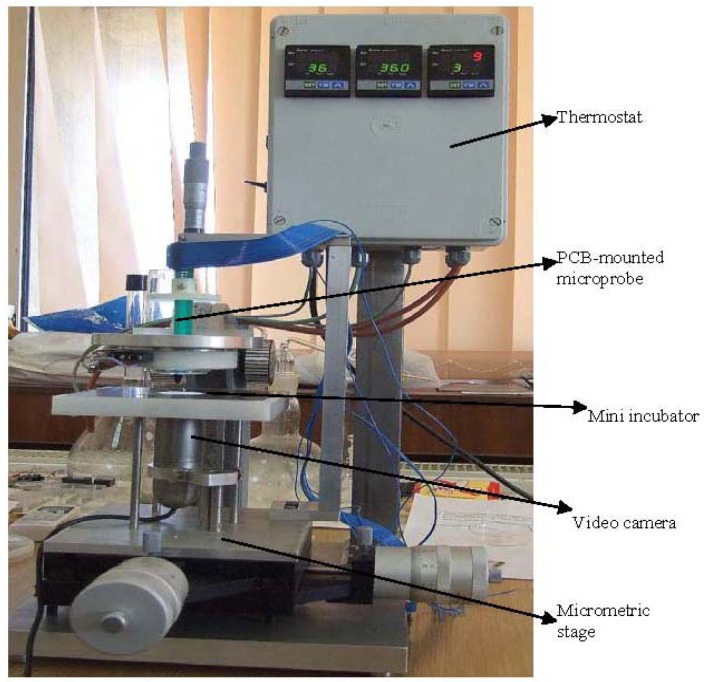
Integrated platform for electrical and optical monitoring of cell cultures.

The developed integrated platform, with the following components, is presented in the Figure 7:

- Micrometric table; video camera; micro-incubator; cell culture vessel provided with channels for nutrient distribution and exhaust; microprobe with electrodes and sensors; reference electrode; graphical interface with electronic modules and computer; video interface for optical monitoring of cells and data acquisition.

**Figure 8.**
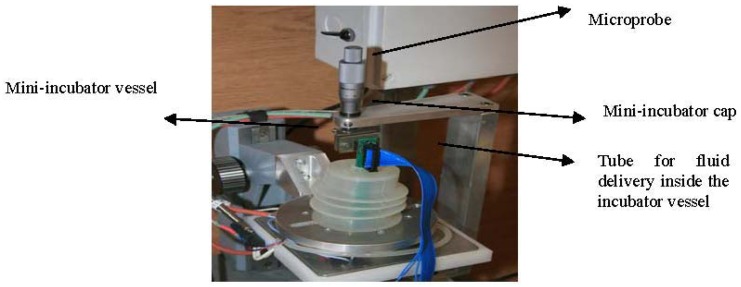
Mini-incubator for monitoring of cell cultures.

The part that we call mini-incubator (presented in Figure 8) contains a special glass vessel of 4 cm in diameter and a special polymeric cap. The glass vessel is thin and as much as possible transparent, allowing a good resolution of pictures taken by a video camera placed underneath (Figure 8). The vessel is provided with two inlets and one outlet connected to the fluidic system (tubes and pumps) for circulation of needed liquids inside the vessel. The fluid circulation was tested in connection with the temperature control inside the incubator. The special polymeric cap is built to be fixed on the glass vessel and has an opening on the top side for microprobe insertion inside. The cap is manufactured to have a very flexible and extendable part allowing the positioning and alignment of the microprobe on Z direction. The vessel and the cap can be sterilized both in temperature (>100 °C) and in chemical solutions without damages or deformations.

### 3.2. Electrical Testing

#### 3.2.1. Electrodes Testing

**Figure 9.**
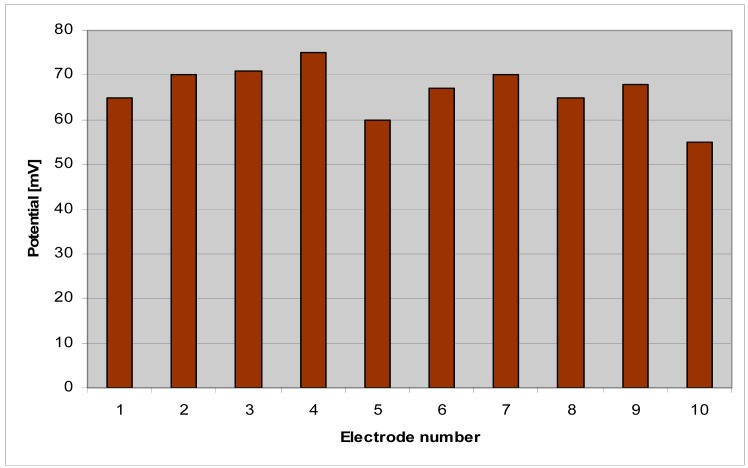
Potential of the 10 electrodes in pH 7.4 buffer solution.

Electrical testing of the 10 electrodes has been performed in buffer solution of pH 7.4 similar to that of cells culture media. The ten electrode potentials are presented in Figure 9.

The microprobe tests have been performed in HaCaT cell culture. The testing step has the role to proving the functionality of the platform in terms of electrical parameters and optical monitoring and data acquisition. The platform was designed to provide electrical information about the cells' potential, impedance, temperature and pH and optical information by storage of cell culture pictures in real time. The choice of HaCaT cells, provided by the Victor Babes Institute, for platform demonstration and testing is due to their resistance under *in vitro* conditions and easy proliferation [13].

Two types of electrodes on top of the microprobe tip have been used: (a) Pt electrodes patterned on a silicon substrate according to the technological steps described in paragraph 2, Figure 1; and (b) Pt electrodes patterned on silicon + SiO_2_, micromachined by laser ablation. The second version comes from the necessity to find methods to decrease the electrode potential. With the structuring of the substrate, the electrode area becomes much larger and the conductivity become higher. Electrical connections, electrical and optical data acquisition, mini-incubator monitoring (temperature, humidity and CO_2_), the microfludic connections and pumping of media inside the incubator have been important achievements of the work of making the platform a useful tool for different applications in cell culture monitoring.

**Figure 10.**
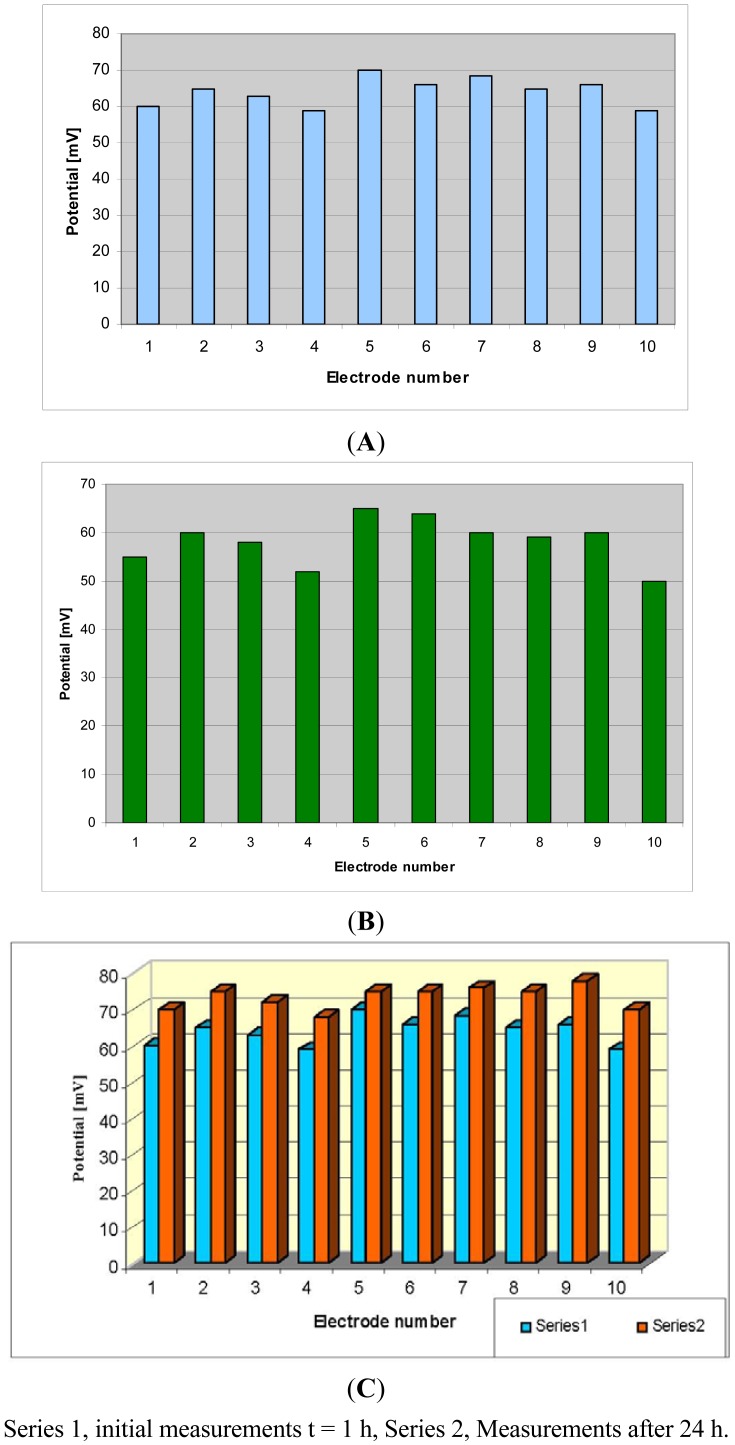
Potential measurement of HaCaT cell culture by using ten (**A**) Pt electrodes on silicon; (**B**) laser nanostructured Pt electrodes on silicon; (**C**) Pt electrode on silicon after 24 h of experiment.

The potential measurement [20] on HACAT cells culture hosted in the micro-incubator at 37 °C by using Pt electrodes on silicon substrate indicates higher values compared (Figure 10(A)) with potential measured with Pt electrodes on nanostructured SiO_2_ substrate (Figure 10(B)). This is a demonstration of the assumption that the electrode impedance is decreasing if the contact area with the environment is increasing.

The comparison between the potential of the HaCaT cell culture measured at the beginning of the experiment using Pt electrodes on silicon and after 24 h growing inside the mini-incubator is presented in Figure 10(C). The increase of the potential after 24 h is an indication of the cell growth process, as the cells have insulating properties and their growth increases the electrical resistance of the medium.

#### 3.2.2. Temperature Sensor Testing

**Figure 11.**
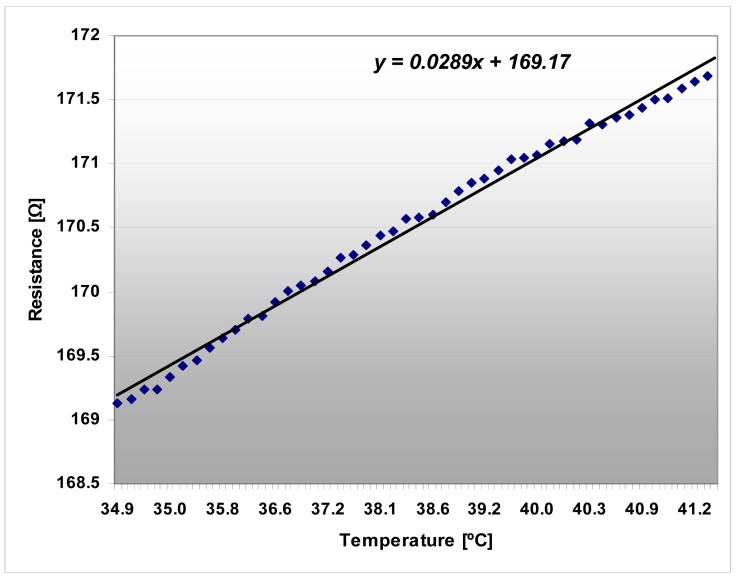
Temperature sensor diagram, resistance *versus* temperature in a water based solution.

The platinum resistor acting as temperature sensor has been calibrated in aqueous solution. The sensor proved its stability for months, with no drift. The precision of the sensor is ±0.2 °C. The testing of the temperature sensors in a thermostated bath indicated their linear characteristics that are presented in Figure 11.

#### 3.2.3. pH Sensor Measurement

**Figure 12.**
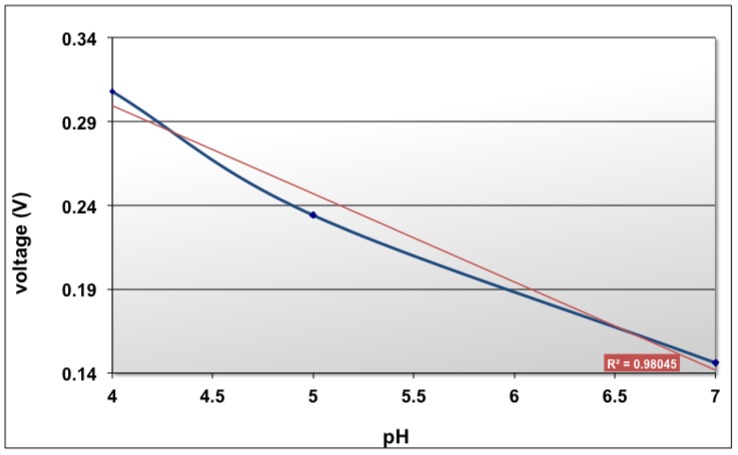
Diagram of voltage *versus* pH.

pH is defined as -log [H+], so we can say that a pH sensor measures and is sensitive to hydrogen ions. pH is characterized by a potential measurement between the working electrode and the reference electrode at zero current. The pH working electrode, which is a Ti/Pt electrode deposited with polyaniline as described in paragraph 2.3 was measured against the reference electrode and a variation of ∼55 mV/pH with the precision of ±5 mV has been obtained (Figure 12).

**Figure 13.**
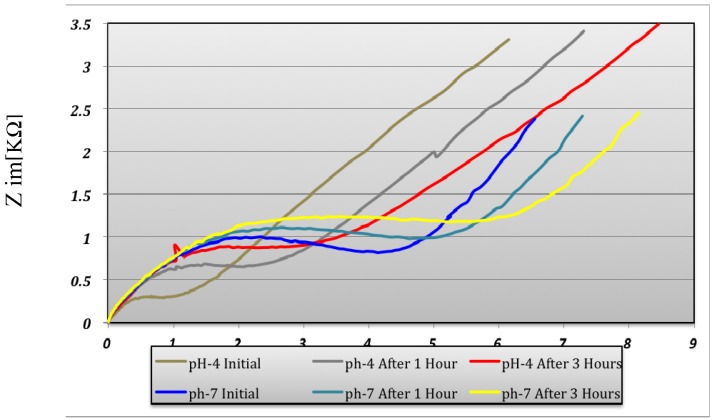
Impedance of Ti/Pt deposited with polyaniline sensor.

At pH 4 the predominant polyaniline state is leucoemeraldine. At pH 7 the predominant state is emeraldine. The transformation of the polyaniline from one state into another state is due to the redox reaction taking place at the solution interface. The polyaniline conductive measurements indicated a variation of conductivity depending on the pH environment (Figure 13).

**Figure 14.**
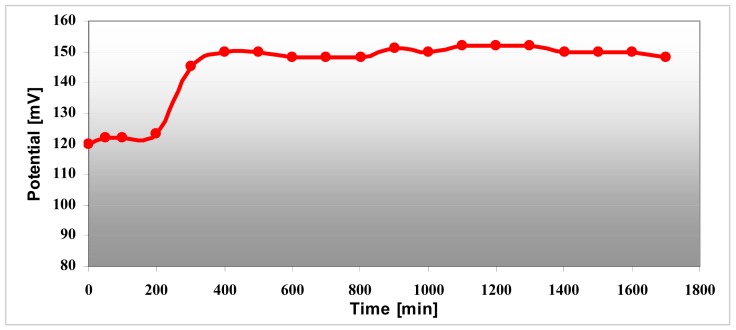
pH sensor diagram, voltage *versus* time in pH 7 buffer solution.

The conductivity increases in strong acid medium like pH 4 compared with pH 7 medium and is maximum at pH 1 and remains constant at this maximum value. Polyaniline variation depends on the pH environment and that way the sensitivity as a pH sensor is demonstrated. The conductivity of the electrode changes over three hours both in pH 4 and pH 7 as can be seen in Figure 13. The pH sensor become more stable after ∼three hours and it provided reproducible results over several days. The interpretation of this result is that the polyaniline suffers a stabilization process when it changes from one state to another state. This remark has been proven by the pH measurement for 30 hours in pH 7 buffer solution (Figure 14). After 200 minutes, the pH sensor shows a good stability and the measured voltage was constantly around 150 mV ± 5%.

### 3.3. Optical Testing

**Figure 15.**
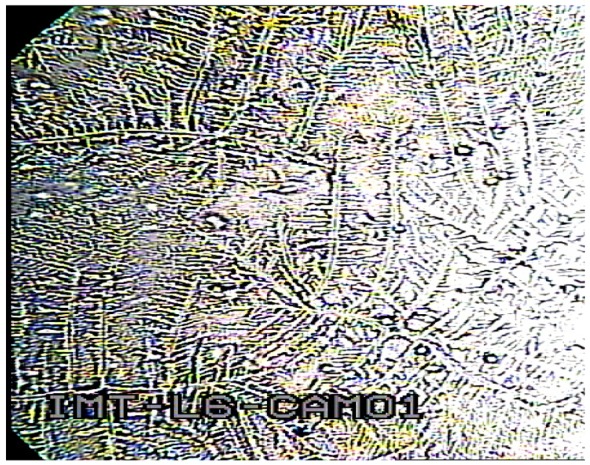
Picture of HACAT cell culture in the mini-incubator after 50 h of culture (×100 magnification).

The video camera, USB connected with the computer, allows obtaining real-time pictures of cell cultures inside the micro-incubator. The dedicated software allows the capture of still pictures or video recording of cells. The camera picture of the HACAT cell culture after 50 h of culture inside the mini-incubator is presented in Figure 15.

## 4. Conclusions/Outlook

The novelty of this work can be summarized in the following points:

- fabrication of a multichannel microprobe with 10 bidirectional electrodes, integrated on a 240 μm width silicon shank and individually addressed, by using and comparing two fabrication methods: (1) patterning of Pt electrodes on SiO_2_ substrate and (2) nanostructuring of SiO_2_ substrate and patterning Pt electrodes on top for increasing the electrode area;
- Development of a special platform for *in vitro* measurements including a miniaturized incubator (with temperature control, sterilized environment, cell feeding by fresh media and CO_2_ control), allowing electrical and optical investigations of cells culture.
- Integration of planar technology based sensors (pH electrodes together with temperature sensor, impedimetric sensors and ten bidirectional electrodes) into a small monolithic silicon chip, designed to work in cell culture medium inside an incubator.
- Development of associated electronics for signal conditioning and data acquisition 24/7 days. The precision of the temperature sensors is ±0.2 °C and the sensitivity is 0.5 Ω/°C as for the pH sensor the sensitivity is 53 mV/pH

The proof of concept of an integrated platform with multiple functions in cells culture monitoring has been provided. Achievement of a reconfigurable platform providing the possibility to easy change the “building blocks”, meaning the electrical, optical and microfluidic interfaces, sensors type and functionality, computer interface and data interpretation, according to the needed application.

## References

[1] Ni M., Tong W.H., Choudhury D., Rahim N.A.A., Iliescu C., Yu H. (2009). Cell culture on MEMS platforms: A review. Int. J. Mol. Sci..

[2] Chang J., Yoon S.H., Mofrad M., Lin L. (2011). MEMS-based dynamic cell-to-cell culture platforms using electrochemical surface modifications. J. Micromech. Microeng..

[3] Bellalou J., Frachon E., Longin R., Meier A. (2006). Robotized platform for cell cultures in miniature reactor batteries, equiped with a system for real time measurement of cellular turbidity or others optical properties.

[4] Moldovan C., Ilian V., Iosub R., Modreanu M., Dinoiu I., Firtat B., Voitincu C. (2002). Micromachining of a silicon multichannel microprobe for neural electrical activity recording. Sens. Actuators A Phys..

[5] Stieglitz T. (2010). Manufacturing, assembling and packaging of miniaturized neural implants. Microsyst. Technol..

[6] Tekeshi K., Kuniharu T., Takahiro K., Ishida M. Microprobe array based Neural Interface Device.

[7] Neves H.P., Orban G.A., Koudelka-Hep M., Ruther P. Development of Multifunctional Probe Arrays for Cerebral Applications.

[8] Moore E., Rawley O., Wood T., Galvin P. (2009). Monitoring of cell growth *in vitro* using biochips packaged with indium tin oxide sensors. Sens. Actuators B Chem..

[9] Paschero A., McLoughlin E., Moore E.J. Continuous Non-Destructive Monitoring of Cell Health using Impedance Based Interdigitated Electrode Structured Sensors.

[10] Stan I., Moldovan C., Necula D., Codreanu C., Firtat B., Codreanu N., Iosub R., Ontanu F., Stefanescu P.I. Aparatus for monitoring cell cultures.

[11] MacDiarmid A. (2001). Synthetic metals: A novel role for organic polymers. Angew. Chem. Int. Ed..

[12] Moldovan C., Iosub R., Radu C., Moore E., Paschero A., Messina W., Demarchi D., Codreanu C., Necula D., Codreanu N. Sensor System for On-Line Monitoring of Cell Cultures.

[13] Life Technologies site http://www.invitrogen.com/site/us/en/home/References/gibco-cell-culture-basics/cell-culture-environment.html.

[14] Kim H.S., Lee G.M. (2007). Differences in optimal pH and temperature for cell growth and antibody production between two Chinese hamster ovary clones derived from the same parental clone. J Microbiol Biotechnol..

[15] Hwang S.J., Yoon S.K., Koh G.Y., Lee G.M. (2011). Effects of culture temperature and pH on flag-tagged COMP angiopoietin-1 (FCA1) production from recombinant CHO cells: FCA1 aggregation. Appl. Microbiol. Biotechnol..

[16] Naciri M., Darrin Kuystermans D., Al-Rubeai M. (2008). Monitoring pH and dissolved oxygen in mammalian cell culture using optical sensors. Cytotechnology.

[17] Arquint P., Koudelka-Hep M., de Rooij N.F., Bühler H., Morf W.E. (1994). Organic membranes for miniaturized electrochemical sensors: Fabrication of a combined pO_2_, pCO_2_ and pH sensor. J. Electroanal. Chem..

[18] Aarts A.A.A., Neves H.P., Puers R.P., van Hoof C. (2008). An interconnect for out-of-plane assembled biomedical probe arrays. J. Micromech. Microeng..

[19] Schürer N., Köhne A., Schliep V., Barlag K., Goerz G. (1993). Lipid composition and synthesis of HaCaT cells, an immortalized human keratinocyte line, in comparison with normal human adult keratinocytes. Exp. Dermatol..

[20] Biran R., Martin D.C., Tresco P.A. (2005). Neuronal cell loss accompanies the brain tissue response to chronically implanted silicon microelectrode arrays. Exp. Neurol..

